# How Does Sexual Harassment Influence the Female Employee’s Negative Response in a Deluxe Hotel?

**DOI:** 10.3390/ijerph17249537

**Published:** 2020-12-19

**Authors:** Hyo Sun Jung, Hye Hyun Yoon

**Affiliations:** 1 Center for Converging Humanities, Kyung Hee University, Seoul 02447, Korea; chefcook@khu.ac.kr; 2 Department of Culinary Arts and Food Service Management, Kyung Hee University, Seoul 02447, Korea

**Keywords:** sexual harassment, psychological distress, deviant behavior, organizational silence, psychological detachment, female employee, deluxe hotel

## Abstract

Today, organizations face risky legal and financial consequences stemming from a single sexual harassment event. The purpose of this study was to verify that the sexual harassment, as perceived by female employees, significantly affects their levels of psychological distress and workplace deviant behavior to investigate the moderating role of organizational silence and psychological detachment in the causal relationship. First, this study found that perceived sexual harassment has a negative impact on the female employees’ psychological distress and workplace deviant behavior. This study’s results also demonstrated that psychological distress has a positive impact on workplace deviant behavior. Additionally, the influence of perceived sexual harassment on psychological distress increased when the employees’ psychological detachment was weak. Finally, limitations and future research directions are also discussed.

## 1. Introduction

**Figure 1:**
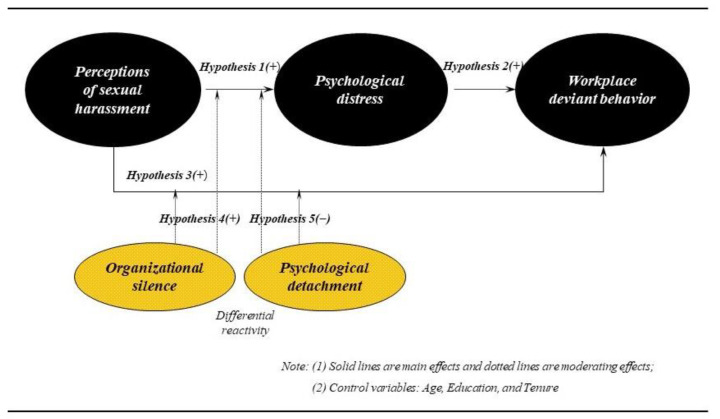
A proposed model of sexual harassment, psychological distress, workplace deviant behavior, organizational silence, and psychological detachment.

Sexual harassment, which is one symptom of social discrimination that occurs when a person becomes subject of unwanted sexual debate, gesture, or action, is presently a critical problem and at the center of attention throughout many organizational environments [1]. The current #MeToo movement raised the public’s awareness of the problem of sexual harassment that women experience within organizations. The movement not only captured the possibility of sexual harassment’s omnipresence within daily workplace functions but also made it clear that people should not be silenced [2]. Today, organizations face risky legal and financial consequences stemming from a single sexual harassment event [3]. Although research on workplace sexual harassment is closely related to psychological health disorders, current longitudinal research on this relationship is scarce. In particular, people working in an environment that involves strong social interaction and stress, such as deluxe hotels, are exposed to excessive psychological distress [4,5]. Due to this type of stressful working environment [6], circumstances of workplace harassment inside such organizations are often specifically silenced [7,8]. For this reason, creating an environment in deluxe hotels where sexual harassment does not occur is a very important factor that can determine the success or failure of the organization. Despite this importance, there is scarce prior study related to this issue. Therefore, this study examines whether the perception of sexual harassment among female employees causes psychological distress and workplace deviant behavior, as well as investigates the moderating role of organizational silence and psychological detachment in the causal relationship (Figure 1).

## 2. Literature Review and Conceptual Model

### 2.1. Sexual Harassment in the Hospitality Sector

Although there is an insufficient amount of academic research regarding sexual harassment in the hospitality industry, the topic has long been a subject of interest within the industry and by researchers. According to Eller [9], the extent of sexual harassment in the hotel industry is not completely clear, as hotel employees experience relatively more cases of sexual harassment than other general workers, with most of the harassment coming from coworkers. Gilbert et al. [10] stated that, among diverse types of harassment, sexual harassment can have a fatal impact on the victim, decrease the individual’s morale, and seriously affect their efficiency. They argued that the problem of sexual harassment is particularly important in the hospitality industry and that the issue should be clearly discussed throughout hiring procedures. In a study that investigated restaurant employees, Weber et al. [11] argue that it is reasonable to say that sexual harassment is more widespread in the restaurant industry because circumstances that can be regarded as sexual harassment in other industries are often not treated as sexual harassment in the restaurant industry. Theocharous and Philaretou [12] believe that many cases of sexual harassment remain unreported in the hospitality industry. According to their study, sexual harassment not only causes direct financial threat and loss to the victims, female employees, but also seriously damages their future career, such as delaying promotion and loss of income. Yusuf and Anuar [13] suggest that inhumane relationships with coworkers or supervisors, as well as aggressive words from them, are the most frequently observed conflict in the hotel industry. Vettori and Nicolaides [14] introduced the peril of sexual harassment in the hospitality industry and suggested policies that managers should provide to maintain a safe and ethical working environment. Li et al. [7] cited that employees’ perceived sexual harassment negatively influences their customer-oriented service behavior and its influence increases when the interpersonal relationship is sensitive. 

### 2.2. Model Development and Hypotheses

#### 2.2.1. Relationship between Sexual Harassment and Psychological Distress

Sexual harassment is unwanted sex-related behavior inside an organization, which makes the victim feel offended, exceeds the individual’s coping resource, and/or threatens the individual’s well-being [15]. Here, not only is unwanted physical behavior included but also unwelcomed verbal and nonverbal sexual behavior [16]. Psychological distress refers to a psychological condition that is characterized by negative thoughts and feelings related to anxiety, fear, or depression [17]. Many studies exist that investigate perceived sexual harassment in organizations and psychological distress. Richman et al. [18] displayed that sexual harassment can exceed the coping resource of the subject of harassment and cause negative psychological behavior. Willness et al. [19] reported that sexual harassment occurring within an organization can damage physical and psychological health as well as cause posttraumatic stress disorder. Among the studies related to sexual harassment and psychological distress, Nielsen and Einarsen (2012) stated that sexual harassment perceived in an organization contributes to the psychological distress of female employees and further argue that organizational measures on this issue can relieve the mental distress. Nielsen et al. [20] showed that all types of harassment occurring in the workplace, including sexual harassment, increase the members’ psychological distress. In a study that used college students as the sample population, McGinnley et al. [21] demonstrated that psychological distress increases as a result of sexual harassment and that the experience is highly likely to keep affecting the victim into the future. Kim et al. [22] reported that sexual harassment experienced by female soldiers in the army negatively impacts their mental health and its influence persisted for a considerable amount of time after their discharge from duty. Wolff et al. [23] explained that sexual harassment can pose a negative influence on health and showed that sexual harassment also positively affects the symptoms of psychological distress, such as depression and anger. O’Neil et al. [24] argued that sexual harassment and other types of sexual violence that occur inside the workplace provide psychological stress to the victims even after removal of the threat. Martinko et al. [25] also suggested abusive supervising as a cause of psychological distress among subordinate employees. Based on these prior studies and further empirical evidence, this study proposes that sexual harassment increases employees’ psychological distress as follows:

**Hypothesis 1** **(H1).** *Perception of sexual harassment positively influences employees’ psychological distress*.

#### 2.2.2. Relationship between Psychological Distress and Workplace Deviant Behavior

Deviant behavior is voluntary action that violates serious organizational norms by which well-being and performance of both the organization and its members are threatened [26]. Spector and Fox [27] stated that because work-related pain or stress causes negative emotion, it induces the members to commit actions that harm the organization itself. Omar et al. [28] said that stress experienced inside an organization, including negative emotions, such as frustration and irritation, positively affects the members’ deviant behavior. Many studies demonstrated that psychological pain, or stress experienced in a work environment, is the major cause of diverse types of deviant behavior [29,30]. Nasurdin et al. [31] reported that strong psychological distress increases the possibility that employees become tense, take impulsive action, and show less tolerance towards other people, which is a demonstrated form of deviation. Similarly, Vigoda [32] argued that work-related pain induces employees’ aggressive behavior. Saleem et al. [33] explained how psychological distress coming from abusive supervisors makes the employees leave the organization. Roxana [34] described how stress factors in workplace are controlled by the environment inside the organization and that the pain or stress linked to the members’ behavior acts against the organization. Aube et al. [35] suggested that psychological well-being, which is an opposite concept of psychological distress, decreases the employees’ counterproductive behavior and that existence of the counterproductive behavior can damage the employees’ psychological health. Therefore, this study assumes the following:

**Hypothesis 2** **(H2).** *Employees’ psychological distress positively influences their workplace deviant behavior*.

#### 2.2.3. Relationship between Sexual Harassment and Workplace Deviant Behavior

Appelbaum et al. [36] stated several reasons why a member of an organization deviates and that sexual harassment is one of the important causes of such behavior. Popovich and Warren [3] argued that sexual harassment and organizational counterproductive behavior is an inseparable relationship, and that sexual harassment is the most fundamental motivation for inducing counterproductive behavior in an organization. Ahmad and Omar [37] reported that rude physical contact from a boss, which is not sexual harassment, increases the possibility that the employee acts in a manner that does harm to the organization. Tangem [38] cited sexual harassment as one of the most important reasons for unproductive workplace behavior and that it causes the strongest counterproductive behavior among female employees. Solakoglu et al. [39] explained that the experience of sexual abuse is strongly related to the possibility of being involved in most types of the deviant behaviors. Zhu et al. [40] demonstrated that sexual harassment in workplace has a strong and positive relationship with deviation in the workplace and further argued that the relationship strengthens when depression occurs. Similarly, Merkin and Shah [41] found that respondents who experienced sexual harassment had a higher turnover intention and rate of absenteeism compared to the respondents without such an experience. Salman et al. [42] suggested that sexual harassment and turnover intention are closely related. Therefore, the following hypothesis is proposed:

**Hypothesis 3** **(H3).** *Perceptions of sexual harassment positively influence workplace deviant behavior*.

#### 2.2.4. Moderating Role of Organizational Silence and Psychological Detachment

Organizational silence makes some employees extremely indifferent and unconcerned. Therefore, indifferent employees mean those who are uninterested in their work, employer, or quality of the work [43,44]. Psychological detachment refers to the work-related experience of when the individual is psychologically “switched off” [45]. No prior study investigates organizational silence and psychological detachment as a variable that moderates negative influence from perceived sexual harassment on employees. Among the studies related to the moderating role of organizational silence, Fernando and Prasad [2] described the harmful effect of a silencing atmosphere with evidence that the silencing organizational atmosphere, which makes victims unable to reveal their dissatisfaction, prevents the sexual harassment from being expressed outside of the organization. Elçi et al. [46] showed that organizational silence, or the atmosphere that allows harassment, increases employee turnover intention. Jain [47] investigated factors that cause silence from an aspect of interpersonal relationship and reported that the silence increases when a vertical hierarchical relationship with a supervisor continues. Zahed [48] argued that social harassment that employees experience from coworkers or a supervisor in an organization causes employee silence within the organization. Huang et al. [49] also explained how a negative relationship with an impersonal and rude supervisor is one of the important causes of employee silence. Mao et al. [50] demonstrated that organizational silence caused low performance and low organizational citizenship behavior, which eventually made highly unproductive behavior predictable. Based on these results, this study assumed that the negative effect of the employees’ perceived sexual harassment on their psychological distress and counterproductive behavior increases when it is accompanied by organizational silence.

**Hypothesis 4** **(H4).** *Organizational silence moderates the effect of sexual harassment on psychological distress and workplace deviant behavior*.

Among the studies related to the moderating role of psychological detachment, Vogel and Mitchell [51] argued that the positive influence of a supervisor’s abusive behavior on turnover intention, not the perceived sexual harassment, decreases as a result of employee psychological detachment. Burris et al. [52] reported that psychological detachment from the organization diminishes the possibility of leaving the organization. Sonnentag et al. [53] explained that psychological detachment experienced in an organization is important because it induces low levels of psychological fatigue and creates time for recovery. Safstrom and Hartig [54] found that psychological detachment plays an intervening role between work requirement and life satisfaction. Chen et al. [55] cited psychological detachment as a negative influence on counterproductive behavior and mediates the influence of work demand on counterproductive behavior. Tong et al. [56] demonstrated that psychological detachment experienced inside an organization becomes one of the stress factors that negatively influences employees’ counterproductive behavior and reduces the harmful effect of psychological stress factors on employees’ performance and well-being. Empirical evidence proves that increased experience of psychological detachment diminishes stress response, such as job burnout [57]. Based on these results, this study assumed that the effect of employees’ perceived sexual harassment on psychological distress and deviant behavior decreases when there is appropriate psychological detachment.

**Hypothesis 5** **(H5).** *Psychological detachment moderates the effect of sexual harassment on psychological distress and workplace deviant behavior*.

## 3. Research Methodology

### 3.1. Sample and Data Collection

The sample for this study consisted of female employees working in deluxe hotels (five-star hotels) located in Seoul, South Korea. Among 22 deluxe hotels located in Seoul, the researcher chose 10 of the hotels that agreed to participate in the survey. The sample was restricted to female employees working in the food and beverage department because many previous studies reported that women are relatively more exposed to sexual harassment situations than men. After the researcher provided sufficient explanation of the purpose and methodology of the study, the subjects, who voluntarily decided to participate in the survey, wrote answers to the questionnaire on a separately prepared space. The researcher reminded responders that the collected data will be used for research purposes only and that it will remain confidential. For even distribution of the extracted samples among the hotels, 50 copies of the questionnaire were distributed to each of the 10 hotels, utilizing the convenience sampling method. At each meeting, the participants received a five-dollar coffee coupon for completing the survey. A total of 350 questionnaires were distributed, among which 312 copies were received. We conducted final analysis on 295 responses (84.29%). Prior to the main analysis, the researcher implemented data screening and analyzed descriptive statistics to verify whether the normality assumption was satisfied. The mean age of the employees was 29.47 (±6.24) years, and 47.7% were between 21 and 29 years old. Additionally, their education levels were found as primarily college (45.3%) and university degree (52.7%), and 62.4% worked in a deluxe hotel for less than 10 years. In addition, job position was indicated as part-time employee (21.8%) and full-time employee (78.2%).

### 3.2. Instrument Development

The questionnaire consisted of six parts. The first part contained questions about the participants’ demographic information (e.g., age, education, and tenure. The second part requested employees to rate their overall perception of sexual harassment. To measure employees’ perceptions of sexual harassment, this study utilized an adapted multi-item scales by Fitzgerald et al. [58] and a modified set from Li et al. [7]. To measure each item, the researcher utilized 6 items and a 7-point scale: “How much do you agree or disagree with these statements?” (1: strongly disagree to 7: strongly agree). The third and fourth parts of the survey focused on employees’ psychological distress and workplace deviant behavior. The researcher used 4 items based on those developed by Kessler et al. [59] and Birkeland et al. [60] to measure employees’ psychological distress, while measuring workplace deviant behavior with 4 items developed by Bennett and Robinson [61]. In addition, the researcher measured organizational silence, which was used as a moderating variable in this study, with four questions based on Dasci and Cemaloglu [62]. The researcher measured the employees’ psychological detachment with four items based on those developed by Sonnentag and Bayer [45] and Sonnentag and Kruel [63]. For all measurement items, the questionnaire, originally written in English by Brislin [64], was translated into Korean, and two bilingual experts reverse-translated the questionnaire into English. Afterward, they examined whether the meaning of the measurement questions differed between the two language versions three times. Then, the researcher implemented the main survey after checking for any ambiguity in the survey questions through a preliminary test. 

### 3.3. Data Analysis

We utilized the SPSS program (Version16, SPSS Inc., Chicago, IL, USA) for demographic analysis, reliability analysis, and correlation analysis of the measurement items. In order to examine the validity of the measurement items, the researcher employed the AMOS program. The SPSS program analyzed the demographic characteristics of the respondent, descriptive statistics, reliability, and correlation. The researcher then applied the two-step approach using AMOS, first assessing the fitness of the measurement model, then the entire model was considered [65]. Next, the researcher conducted confirmatory factor analysis (CFA) to test the validity of the measurement item, with the structural equation modeling (SEM) used to check the hypothesis. A multi-group analysis (MGA) tested the moderating role of organizational silence and psychological detachment. 

## 4. Results

### 4.1. Measurement Model

In the analysis results, the Skewness ranged between −0.525 and +0.414, and the Kutosis ranged between −0.203 and −0.759, which implies that the normality is satisfied. All measurement items had a Z-score between −3 and +3, indicating the absence of a univariate outlier problem. Kline [66] and Hair et al. [67] reported that Skewness and Kutosis ranging between −3 and +3 allows normal distribution. Therefore, it is proof that the probability of a univariate outlier in this study data is minimal [68]. This study identified convergent validity, discriminant validity, and nominal validity to verify the validity of the measurement items. We examined convergent validity, discriminant validity, and nomological validity to verify construct validity (Table 1 and Table 2). Additionally, we conducted CFA, which is a method of analyzing the structural effectiveness of the collected measurement variables before investigating the causal relationship of a developed theoretical model. As shown in Table 1, Cronbach’s alpha ranged between 0.93 and 0.96, and CCR exceeded 0.80 in all cases, indicating satisfaction of the approval criteria [69,70]. Furthermore, the standardized coefficient of all measurement items was over 0.80, and the result was significant at 0.001 level [71]. The overall fitness of the model was excellent (χ^2^ = 424.72; df = 220; χ^2^/df = 2.01; GFI = 0.89; CFI = 0.98). The square root of the coefficient of all measurement items (0.06~0.66) was smaller than AVE (0.79~0.85). AVE showed a higher value than ASV and MSV, thus confirming discriminant validity.

### 4.2. Structural Equation Modeling

We analyzed the relationship among the variables assumed in this study using SEM. Table 3 presents the standardized path coefficient of the relationship of the structural equation model and *t*-value [67,72]. The structural model fit was good (χ^2^ = 210.66; df = 87; χ^2^/df = 2.42; GFI = 0.91; NFI = 0.96; CFI = 0.98; RMR = 0.08; RMSEA = 0.07). Hypothesis 1, which assumed positive relationship between the perceived sexual harassment and psychological distress, was supported (β = 0.26; *t* = 4.30; *p* < 0.001). Hypothesis 3 was also supported, as the perceived sexual harassment showed positive impact on workplace deviant behavior (β = 0.48; *t* = 8.56; *p* < 0.001). Hypothesis 2, which assumed that psychological distress gives positive influence on workplace deviant behavior, was supported as well (β = 0.18; *t* = 3.41; *p* < 0.001). This result demonstrates that people who perceived sexual harassment inside an organization show psychological distress, which consequently increases the possibility of employee action that does harm to the organization. In consideration of Hypotheses 1 and 3, the researcher additionally tested the indirect effects of perceived sexual harassment on workplace deviant behavior by the *Bootstrap* and *Sobel* test. In the test results, the perceived sexual harassment showed a partial mediating effect on workplace deviant behavior through psychological distress (Point estimate = 0.05, *p* < 0.05, Z–score = 2.87). The result proves the importance of the role that psychological distress plays in the impact of perceived sexual harassment on workplace deviant behavior. Furthermore, we conducted multi-group analysis to examine whether the atmosphere of organizational silence and psychological detachment reduces the negative influence of the perceived sexual harassment. When using organizational silence as a moderating variable, no moderating role of organizational silence was observed in the causal relationship between sexual harassment and psychological distress and workplace deviant behavior. Therefore, Hypothesis 4 was rejected (Table 4). However, the influence of perceived sexual harassment on psychological distress showed different results in the case of psychological detachment. The weaker the psychological detachment was, the stronger the influence of the perceived sexual harassment on psychological distress. Therefore, Hypothesis 5 was partially supported (Table 5).

## 5. Discussion and Implications

### 5.1. Discussion and Theoretical Implications

This study examined the effect of sexual harassment perceived by female employees in deluxe hotels on their psychological distress and workplace deviant behavior, as well as investigated the moderating effect of organizational silence and psychological detachment that diminishes its negative influence. First, this study found that perceived sexual harassment has a negative impact on the female employees’ psychological distress and workplace deviant behavior. This result is consistent with the existing literature [3,19,24,40]. It implies that employees undergo psychological distress when they experience sexual harassment inside an organization, which further increases the possibility of conducting deviant behavior within the workplace. This study’s results also demonstrated that psychological distress has a positive impact on workplace deviant behavior, confirming the outcome of earlier studies [28,31]. The employees who underwent psychological distress were more likely to voluntarily exhibit behaviors contrary to those benefiting the organization. Furthermore, the influence of perceived sexual harassment on psychological distress increased when the employees’ psychological detachment was weak [51,52]. This result implies that psychological detachment, where work-related experience is switched off, relieves the negative influence of sexual harassment. 

This study systematically examined sexual harassment that female employees in deluxe hotels perceive and investigated the organic causal relationship between psychological distress and deviant behavior. The purpose was to provide a theoretical base for research on sexual harassment in deluxe hotels. Although relevant studies used general companies as a sample, there is a limited number of sexual harassment-related studies that investigate psychological distress or deviant behavior among female employees in deluxe hotels. In this context, this study has significant meaning as an early-stage study that examines the variable of sexual harassment, which exerts absolute influence on the psychological distress and negative deviant behavior from diverse aspects. In particular, employees working in deluxe hotels have intense psychological distress due to interaction with diverse members of the organization. The emotional pain and deviant behavior experienced by employees is of central importance because they are factors that determine a hotel’s competitiveness and job effectiveness. Thus far, however, organizations continue to consider sexual harassment as the deviant behavior of certain individuals with personal problems. With consideration of the current situation, this study has a significant meaning for the observation of the negative influence of sexual harassment. This study also has significant meaning as early research on new variables that can relieve the negative influence of sexual harassment by examining the moderating role of organizational silence and psychological detachment, which have not been examined thus far.

Unethical behavior that occurs in a workplace, such as sexual harassment, is an important problem that not only decreases the quality of life of an individual employee but also is directly connected to organizational performance. In regard to this point, this study provides the following practical implications. An organization should recognize the harmful impact of sexual harassment and make arduous efforts to prevent and solve the problem. The study showed that psychological distress increased, and deviant behavior is largely affected when employees perceived sexual harassment in an organization. For effective management of sexual harassment at an organization level, employers should provide victim-oriented policies so that victims can report an event and testify without fear of retaliation from assailants, including supervisors and coworkers. All institutionalized factors that can cause sexual harassment in an organization’s work environment should be sought out and removed. Through an internal report system, these behaviors should be criminalized, and strict guidelines put in place. Specific guidelines include the following: establishing clear standards that show an example of sexual harassment, flagging the danger of sexual harassment with a prevention videos or promotion program, and creating a department in charge of managing a sexual harassment problem. Considering that psychological detachment can relieve the negative influence of sexual harassment to a certain point, organizations need to provide employees with an opportunity for psychological detachment that focuses on a recharging and recovering experience. With a chance to recharge personal resources, the victim will be able to escape from the psychological distress that results from sexual harassment. Instead of simply being away from the work environment, or not performing work, subjective factors are needed that can make the individual feel that they are able to detach from the environment itself [73]. Organizations should encourage not only psychological detachment in the employee’s personal life outside of the workplace, such as taking leave or a vacation, but also psychological detachment and recovery in work-related spaces inside the organization, such as break times and lunch [74]. Based on these results, the researcher, with a long-term perspective, expects this study to contribute to discovering ways to establish practical policies in deluxe hotels regarding sexual harassment. 

### 5.2. Limitations and Future Research

This study has several limitations. First, this study’s results come from a sample that consisted of Korean employees, which poses a restriction on the generalization of the study’s results. Second, the self-report method was used to measure the perception of sexual harassment, psychological distress, workplace deviant behavior, and organizational silence, and psychological detachment, which could have made the subjects respond in a manner they believed to be desirable. Therefore, more objective measurement tools should be chosen in future studies. Third, workplace deviant behavior was used as a final dependent variable in this study. In the future, variables that reflect the organizational performance should be employed. Fourth, although organizational silence and psychological detachment were moderating variables in this study, exploration of new variables that can moderate the negative influence of sexual harassment is also necessary. Additional research that investigates the relationship between diverse independent variables that can affect the organizational environment where sexual harassment can occur, and the dependent variables are necessary in the future. 

## Figures and Tables

**Table 1:** Confirmatory factor analysis and reliability analysis.

Construct	Standardized Estimate	*t*-Value	Corrected Item-Total Correlation	CCR ^a^ Cronbach’s Alpha
Sexual harassment				0.93
SH_1_	0.89	Fixed ***	0.84	0.96
SH_2_	0.92	25.09	0.89	
SH_3_	0.92	25.05	0.86	
SH_4_	0.90	24.01	0.87	
SH_5_	0.92	24.41	0.90	
SH_6_	0.89	22.39	0.87	
Psychological distress				0.87
PS_1_	0.87	fixed	0.82	0.93
PS_2_	0.94	23.51	0.89	
PS_3_	0.90	21.62	0.87	
PS_4_	0.84	19.09	0.79	
Workplace deviant behavior				0.91
WDB_1_	0.90	fixed	0.87	0.95
WDB_2_	0.91	25.12	0.89	
WDB_3_	0.94	27.51	0.92	
WDB_4_	0.92	25.76	0.89	
WDB_5_	0.87	22.70	0.86	
Organizational silence				0.92
OS_1_	0.94	fixed	0.90	0.95
OS_2_	0.95	34.31	0.92	
OS_3_	0.93	31.72	0.91	
OS_4_	0.83	22.02	0.80	
Psychological detachment				0.89
PD_1_	0.93	fixed	0.89	0.95
PD_2_	0.94	29.63	0.90	
PD_3_	0.95	30.54	0.91	
PD_4_	0.86	23.30	0.83	

Note: ^a^ CCR = composite construct reliability; Standardized estimate = β-value; χ^2^ = 424.72 (df = 220) *p* < 0.001; χ^2/^df = 1.93; Goodness of Fit Index (GFI) = 0.89; Normed Fit Index (NFI) = 0.95; Tucker Lewis Index (TLI) = 0.97; Comparative Fit Index (CFI) = 0.98; Incremental Fit Index (IFI) = 0.98; Root Square Error of Approximation (RMSEA) = 0.06; Root Mean Square Residual (RMR) = 0.08; *** *p* <.001.

**Table 2:** Correlation analysis and discriminant validity tests.

Construct	1	2	3	4	5	AVE	ASV	MSV	Mean ± SD
1. Sexual harassment	1	*0.04*	*0.13*	*0.06*	*0.10*	0.82	0.16	0.26	3.24 ± 1.37
2. Psychological distress	**0.20**	1	*0.05*	*0.51*	*0.01*	0.79	0.27	0.71	2.61 ± 1.41
3. Workplace deviantbehavior	**0.37**	**0.23**	1	*0.16*	*0.10*	0.83	0.15	0.26	3.65 ± 1.47
4. Organizational silence	**0.25**	**0.66**	**0.40**	1	*0.06*	0.83	0.25	0.71	2.81 ± 1.38
5. Psychological detachment	**−0.32**	**−0.01**	**−0.32**	**−0.25**	1	0.85	0.12	0.16	3.95 ± 1.52

Note: AVE = Average variance extracted; ASV = Average shared variance; MSV = Maximum shared variance; **Boldface type** are significant at *p* < 0.05; *Italic type* are presented in squared correlation; SD = Standard Deviation; All items were measured on a 7-point Likert scale from 1-strongly disagree to 7-strongly agree.

**Table 3:** Structural estimates model.

Hypothesized Path (Stated as Alternative Hypothesis)	Standardized Coefficients	*t*-Value	Results
H1(+) Sexual harassment → Psychological distress	+0.26	+4.30 ***	Supported
H2(+) Psychological distress → Workplace deviant behavior	+0.18	+3.41 ***	Supported
H3(+) Sexual harassment → Workplace deviant behavior	+0.48	+8.56 ***	Supported
Goodness-of-fit statistics	χ^2^_(df = 87)_ = 210.66 (*p* < 0.001) χ^2/^df = 2.42		
	GFI = 0.91 NFI = 0.96		
	CFI = 0.98		
	RMR = 0.08		
	RMSEA = 0.07		

Note: (1) GFI = Goodness of Fit Index; NFI = Normed Fit Index; CFI = Comparative Fit Index; RMR **=** Root Mean Square Residual; RMSEA = Root Mean Square Error of Approximation; *** *p* < 0.001. (2) Mediating role of psychological distress. Indirect effect: Sexual harassment→psychological distress → Workplace deviant behavior Point estimate: +0.05 *; bias-corrected bootstrap 95% CI: 0.02(LL); 0.01(UL) *Aroian* version of the *Sobel* test: Z = 2.87 *.

**Table 4:** Moderating effects of organizational silence.

	High− Organizational Silence (*n* = 152)		Low− organizational silence (*n* = 143)		Unconstrained Model Chi-Square (df = 174)	Constrained Model Chi-Square (df = 275)	∆χ^2^ (df = 1)
	Standardized Coefficients	*t*-Value	Standardized Coefficients	*t*-Value			
Sexual harassment → Psychological distress	0.12	1.45 ^ns^	−0.08	−1.01 ^ns^	312.47	314.89	2.42 ^ns^
Sexual harassment → Workplace deviant behavior	0.43	5.76 ***	0.43	5.07 ***	312.47	313.10	0.63 ^ns^

Note: χ^2/^df = 1.79; GFI = 0.89; NFI = 0.93; TLI = 0.96; CFI = 0.97; IFI = 0.97; RMSEA = 0.05; *** *p* < 0.001; ^ns^ Not significant.

**Table 5:** Moderating effects of employees’ psychological detachment.

	High− Psychological Detachment (*n* = 135)		Low− Psychological Detachment (*n* = 160)		Unconstrained Model Chi-Square (df = 174)	Constrained Model Chi-Square (df = 175)	∆χ^2^ (df = 1)
	Standardized Coefficients	*t*-Value	Standardized Coefficients	*t*-Value			
Sexual harassment → Psychological distress	0.19	2.32 *	0.57	7.19 ***	287.47	311.37	23.90 *
Sexual harassment → Workplace deviant behavior	0.30	6.08 ***	0.29	2.99 ***	287.47	287.58	0.11 ^ns^

Note: χ^2/^df = 1.65; GFI = 0.89; NFI = 0.94; TLI = 0.97; CFI = 0.97; IFI = 0.98; RMSEA = 0.04; * *p* < 0.05, *** *p* < 0.001, ^ns^ Not significant.
